# Clinical implications of trichomonads detected in bronchoalveolar fluid by metagenomic next-generation sequencing: a multicenter retrospective study

**DOI:** 10.3389/fcimb.2024.1289231

**Published:** 2024-01-22

**Authors:** Juan Jiang, Yuanyuan Li, Qiong Wang, Huihui Zeng, Wei Yang, Yanhao Wu, Wenzhong Peng, Pinhua Pan, Chengping Hu, Pengbo Deng

**Affiliations:** ^1^ Department of Respiratory Medicine, National Key Clinical Specialty, Branch of National Clinical Research Center for Respiratory Disease, Xiangya Hospital, Central South University, Changsha, China; ^2^ Center of Respiratory Medicine, Xiangya Hospital, Central South University, Changsha, China; ^3^ Clinical Research Center for Respiratory Diseases in Hunan Province, Changsha, China; ^4^ Hunan Engineering Research Center for Intelligent Diagnosis and Treatment of Respiratory Disease, Changsha, China; ^5^ Department of Respiratory and Critical Care Medicine, Zhuzhou Second Hospital, Zhuzhou, China; ^6^ Department of Pulmonary and Critical Care Medicine, The Second Xiangya Hospital, Central South University, Changsha, China

**Keywords:** pulmonary trichomoniasis, trichomonads, metagenomic next-generation sequencing (mNGS), Trichomonas tenax, trichomonas vaginalis

## Abstract

**Background:**

Pulmonary trichomoniasis is considered a neglected disease due to failures in recognizing it, stemming from insensitive microbial methods and a lack of specific clinical features. This study aims to analyze the clinical implications of trichomonads detected in bronchoalveolar lavage fluid (BALF) by metagenomic next-generation sequencing (mNGS).

**Methods:**

This multicenter retrospective study included patients diagnosed with pneumonia, admitted to three tertiary hospitals in China from July 2018 to September 2022, with trichomonads detected in BALF through mNGS. The analysis covered demographics, comorbidities, symptoms, laboratory findings, mNGS results, clinical treatment, and outcomes of these patients.

**Results:**

A total of 17 patients were enrolled, comprising 14 males and 3 females. Trichomonas tenax and Trichomonas vaginalis were detected by mNGS in BALF samples of 15 and 2 patients, respectively. Patients were categorized into two groups based on the presence of risk factors for trichomonad infection, including immunocompromised conditions, uncontrolled diabetes mellitus, oral/periodontal diseases, and aspiration. Among 11 patients with risk factors (Case 1-11), 4 received nitromidazoles as part of comprehensive treatment, achieving a 100% treatment success rate. The remaining 7 patients, who did not receive nitromidazoles, had only one achieving relief after broad-spectrum antimicrobial therapy, resulting in a 14.3% treatment success rate. For the 6 patients without any risk factors for trichomonad infection (Case 12-17), none received nitromidazoles during hospitalization. However, 4 out of these 6 patients (66.7%) eventually recovered.

**Conclusion:**

mNGS proves to be an efficient tool for detecting trichomonads in BALF samples. Comprehensive analysis of clinical features and laboratory indicators is essential to distinguish between infection and colonization of trichomonads. Pulmonary trichomoniasis should not be overlooked when trichomonads are detected in BALF from patients with risk factors.

## Background

Trichomonads are flagellated protozoan parasites often found in the digestive or reproductive systems of many invertebrates and vertebrates. There are four trichomonad species classically recognized as human parasites, including *Trichomonas tenax* (*T. tenax*) in the oral cavity, *Dientamoeba fragilis* and *Pentatrichomonas hominis* in the intestinal tract, and *Trichomonas vaginalis* (*T. vaginalis*) in the genitourinary tract (Maritz et al., 2014). Among them, *T. vaginalis* and *T. tenax* are considered human-specific. *T. vaginalis* is the causative agent of trichomoniasis, one of the most common sexually transmitted diseases worldwide (Mielczarek and Blaszkowska, 2016). *T. tenax*, an obligate symbiont in the human oral cavity which could be pathogenic in some contexts (Hirt, 2019), especially in patients with poor oral hygiene and advanced periodontal diseases. There is a clear connection between *T. tenax* protozoa and periodontitis disease. Previous studies and meta-analyses support that *T. tenax* is involved in the pathophysiology of periodontal diseases (Marty et al., 2017; Eslahi et al., 2021; Martin-Garcia et al., 2022), even though its individual contribution requires further exploration by more studies. Specifically, *T. tenax* has been identified by microscopic and molecular methods in the lower respiratory tract specimens (Mallat et al., 2004).

Pulmonary trichomoniasis, which is caused by infections with trichomonads in the lungs, has been previously reported in only a few cases with risk factors, including immunocompromised conditions, uncontrolled diabetes mellitus, oral/periodontal diseases and aspiration. Most of these cases were infected with either *T. tenax* (Hersh, 1985; Lewis et al., 2003; Mallat et al., 2004) or *T. vaginalis* (Temesvari et al., 2002; Duboucher et al., 2003; Carter and Whithaus, 2008). Wet mount microscopy is widely used and highly specific, but its sensitivity is low in vaginal specimens (Bandea et al., 2013; Leli et al., 2016). Conceivably, it is even more difficult to sensitively detect trichomonads in BALF, which is considered as a dilute form of the composite epithelial lining fluid. While polymerase chain reaction (PCR) assay has been proved to detect trichomonads in both vaginal and BALF specimens in a sensitive and specific manner (Heine et al., 1997; Shipitsyna et al., 2013; Lin et al., 2019), it is not routinely applied for patients with pneumonia, due to the poor accessibility to the PCR assay of trichomonads and a lack of knowledge and vigilance on pulmonary trichomoniasis at present. Therefore, it remains challenging to achieve sensitive and specific detection of trichomonads in BALF, further aggravating the unawareness of trichomonads in the lungs.

Metagenomic next-generation sequencing (mNGS) is a promising microbial detection technology in infectious diseases (Jiang et al., 2021; Chen et al., 2022). Based on high-throughput sequencing, mNGS detects microbial nucleic acids in a variety of specimens and identifies a wide range of pathogens by a single test. Compared with conventional methods, mNGS is unbiased, rapid and highly sensitive, making it a popular microbial test in pulmonary infectious diseases, especially for detecting rare or novel pathogens. However, the utility of mNGS for the detection of trichomonads has not been reported so far.

In this study, we conducted a retrospective summary of the clinical characteristics, treatment processes, and outcomes of patients in whom trichomonads were detected in bronchoalveolar lavage fluid (BALF) using metagenomic next-generation sequencing (mNGS). Our objective was to analyze the potential clinical implications of trichomonad presence. The primary aim of this study is to propose feasible strategies for clinical management when trichomonads are identified in BALF. Additionally, we aim to raise awareness among clinical physicians regarding the potential occurrence of trichomonads, either alone or as coinfecting pathogens, in the context of pulmonary diseases.

## Methods

### Study design and subjects

In this retrospective multicenter study, we sequentially screened patients admitted to three tertiary hospitals in China—Xiangya Hospital, Zhuzhou Second Hospital, and Second Xiangya Hospital—between July 1, 2018, and September 30, 2022. A total of 1,680 patients underwent screening, among whom 17 individuals with trichomonads detected in BALF via mNGS were included. Patients were eligible for enrollment if they met the following criteria: (1) age ≥18 years old; (2) radiological evidence indicative of newly emerged pneumonia on chest computed tomography; (3) detection of trichomonads in BALF samples by mNGS. The study was approved by the Institutional Review Board and Ethics Committee of Xiangya Hospital, Central South University (202305381). The ethics committee/institutional review board waived the requirement of written informed consent for participation from the participants or the participants’ legal guardians/next of kin because this is a retrospective study and all research data were de-identified and anonymously analyzed.

Data regarding demographics, comorbidities, symptoms, laboratory and radiological findings, mNGS results, clinical treatment, and outcomes were extracted from electronic medical records. Lymphopenia was defined as lymphocyte count less than 1.1 × 10^9^/L in peripheral blood (Warny et al., 2018). According to the manufacturers’ instructions, the normal ranges of C-reactive protein, procalcitonin and lactate dehydrogenase were defined as 0-8 mg/L, 0-0.1 ng/mL and 120-250 U/L, respectively.

Given that trichomonads are opportunistic pathogens, risk factors for trichomonad infection at baseline were defined based on published literature on pulmonary trichomoniasis (Leterrier et al., 2012; Lopez-Escamilla et al., 2013; Dybicz et al., 2018; Dong et al., 2019): (1) Primary or secondary immunocompromised conditions (Ramirez et al., 2020), including but not limited to hematologic malignancies, solid tumors, rheumatic diseases, long-term systemic use of corticosteroids, use of immunosuppressive agents, solid organ transplantation and hematopoietic stem cell transplantation. (2) Uncontrolled diabetes mellitus. (3) Presence of oral/periodontal diseases and risk factors for aspiration, including sedative use, drug abuse, alcohol consumption, dementia, traumatic brain injury, stroke, seizures, and any therapeutic or diagnostic procedures involving the upper thorax or esophagus. The third risk factor specifically pertains to *T. tenax* infection in the lungs, given *T. tenax*’s residence in the human oral cavity.

### Sample processing and DNA extraction for mNGS

BALF samples were processed as described in a previous study of our group (Jiang et al., 2021). Briefly, BALF was mixed with lysozyme and 1 g of 0.5-mm glass beads, and then the mixture was attached to a horizontal platform on a vortex mixer and agitated vigorously at 2800–3200 rpm for 30 min. For nucleic acid extraction, 300 mL of supernatant was transferred to a 1.5-mL centrifuge tube. Subsequently, DNA was extracted using the TIANamp Micro DNA kit (Tiangen Biotech) according to standard procedures.

### DNA library preparation and sequencing for mNGS

The DNA library was constructed by DNA fragmentation, end repair and PCR amplification using MGIEasy Cell-free DNA Library Prep Set (MGI Tech). Agilent 2100 (Agilent Technologies) and Qubit 2.0 (Invitrogen) were used as library quality control. The double-stranded DNA library was converted into single-stranded circular DNA using DNA degradation and circularization. The DNA Nanoballs were generated by rolling circle amplification technology. Qualified DNA Nanoballs were loaded on the chip and then performed 20 M 50-bp single-end sequencing on the MGISEQ-2000 sequencing platform (BGI Genomics) (Jeon et al., 2014).

### Bioinformatic analysis for mNGS

High-quality sequencing data were generated by removing low-quality reads, followed by computational subtraction of human host sequences mapped to the human reference genome (hg19) using Burrows-Wheeler Alignment (Li and Durbin, 2010; Schmieder and Edwards, 2011). The remaining high-quality data were classified by simultaneously aligning to Pathogens metagenomics Database (PMDB) to obtain the specific number of reads for a particular microbial species, including bacteria, fungi, viruses and parasites. The classification reference databases were downloaded from NCBI (ftp://ftp.ncbi.nlm.nih.gov/genomes/). RefSeq contains the whole genome sequence of 4,945 viruses, 6,350 bacteria, 1064 fungi related to human infections, and 234 parasites associated with human diseases. The coverage ratio and depth of each microorganism were calculated using BEDTools (Quinlan and Hall, 2010). Clinically significant microbes detected by mNGS were determined based on a comprehensive analysis of the read number, coverage ratio, relative abundance, as well as the clinical, laboratory, and radiological characteristics. The sequencing data presented in the study are deposited in the CNGB Sequence Archive (CNSA) of China National GeneBank DataBase (CNGBdb) repository(https://db.cngb.org/cnsa/), accession number CNP0005055.

### PCR assay for the detection of trichomonads

PCR assay was performed to confirm the existence of trichomonads in BALF according to the standardized protocol. Briefly, the DNA lysates were extracted and purified from BALF samples that were positive for trichomonads identified by mNGS by using QIAamp DNA Mini Kit (QIAGEN, Hilden, Germany) according to manufacturer’s instructions. Two pairs of primers targeting a conserved region of either *T. vaginalis* or *T. tenax* with good sensitivity and specificity were synthesized and utilized according to published literatures (Kikuta et al., 1997; Bandea et al., 2013). The sequences were as follows: *T. vaginalis* Former primer 5’-CGAATGGTATAACGAATGCGAC-3’, and *T. vaginalis* Reverse primer 5’-CAACCTTTCTTGTCAGACAACTTG-3’, with product size of 237 bases (NW_001820796.1:258918-259154 Trichomonas vaginalis G3 1047229024264 genomic scaffold); *T. tenax* Former primer 5’-AGTTCCATCGATGCCATTC-3’, and *T. tenax* Reverse primer 5’-GCATCTAAGGACTTAGACG-3’, with product size of 776 bases in 18S ribosomal RNA region. All primer pairs generated fragments of correct size and specificity as verified by DNA sequencing. For PCR amplification, one nanogram of DNA template was mixed with 22 μL of Super PCR Mix (BGI, Beijing, China) and 1 μL of each primer. The steps of PCR were described as follows: initial denaturation for 5 minutes at 96°C, 35 cycles of 20 seconds at 96°C for denaturation, 30 seconds at 52°C for annealing, 30 seconds at 72°C for elongation, a final elongation step for 10 minutes at 72°C. Agarose gel electrophoresis was then performed to assess PCR product yield. Subsequently, PCR products were purified by magnetic beads-based system (BGI, Beijing, China) according to the manufacturer’s instruction. Purified DNA fragments were further confirmed by DNA sequencing.

## Results

### Baseline clinical characteristics

A total of 17 patients were enrolled in this study, comprising 14 males and 3 females. As depicted in **Table 1**, their ages ranged from 29 to 81 years, with 7 patients having a history of current or former smoking. Five patients reported prior oral or periodontal diseases. The most prevalent comorbidities were high blood pressure and diabetes mellitus. Four patients had a history of using immunosuppressive agents before the disease onset. The commonly reported symptoms included fever, cough, and dyspnea. Patients exhibited an oxygenation index ranging from 73 to 250, indicating severe hypoxemia. Elevated white blood cell counts were observed in 6 out of 17 patients. Lymphopenia was frequently noted, whereas increased eosinophils in peripheral blood were seen in only one patient. Elevated levels of C-reactive protein, procalcitonin, and lactate dehydrogenase were found in 100%, 88.2%, and 76.5% of patients, respectively. All patients displayed radiological abnormalities in bilateral lungs with multifocal lesions. In summary, these patients did not exhibit distinctive symptoms, laboratory, or radiological findings. This suggests difficulty in distinguishing pulmonary trichomoniasis from pneumonia caused by other pathogens based solely on clinical features.

**Table 1:** Baseline clinical characteristics of patients.

Case No.	Gender	Age (years)		Current or former smoker		Oral or periodontal diseases		Comorbidities	Use of immunosuppressive agents		Main symptoms	
1	M	63		No		Yes		Follicular lymphoma	Chemotherapy		Fever, cough and dyspnea	
2	F	29		No		Yes		Kidney transplantation	Corticosteroids and Tacrolimus		Fever and dyspnea	
3	F	50		No		No		AML	Chemotherapy		Fever, dyspnea and fatigue	
4	M	64		Yes		Yes		DM, HBP	No		Fever, cough and dyspnea	
5	M	65		No		No		DM, HBP, CAD	No		Fever, cough and dyspnea	
6	M	72		Yes		No		DM, COPD, HBP	No		Cough and dyspnea	
7	M	69		No		No		DM, HBP	No		Fever and dyspnea	
8	F	68		No		No		DM, HBP, CAD	No		Fever, cough and dyspnea	
9	M	74		Yes		Yes		Lung cancer, COPD	Anti-PD-1 monoclonal antibody		Fever and dyspnea	
10	M	45		No		Yes		Cerebral hemorrhage	No		Fever and dyspnea	
11	M	57		No		No		DM, HBP, CAD	No		Cough and dyspnea	
12	M	81		No		No		HBP	No		Fever	
13	M	70		Yes		No		Chronic bronchitis	No		Cough and dyspnea	
14	M	49		No		No		HBP, CVD	No		Fever and cough	
15	M	53		Yes		No		HBP, CVD	No		Fever	
16	M	75		Yes		No		CAD	No		Cough and dyspnea	
17	M	74		Yes		No		COPD	No		Fever, cough and dyspnea	

F, female; M, male; AML, acute myeloid leukemia; DM, diabetes mellitus; HBP, high blood pressure; CVD, cerebrovascular disease; CAD, coronary artery disease; COPD, chronic obstructive pulmonary disease; PD-1, programmed cell death protein 1.

**Table 1:** (continued).

Case No.	Oxyge-nation index	WBC (×10^9^/L)	Neutrophils (×10^9^/L)		Lymphocytes (×10^9^/L)		Eosinophils (×10^9^/L)			CRP (mg/L)		Procalcitonin (ng/mL)		LDH (U/L)	Radio-logical abnormalities	
															Bilateral lungs	Multifocal lesions
1	250	4.2	3.24		0.2		<0.01			145		4.30		410	Yes	Yes
2	188	8.89	7.83		0.25		0.08			114		8.83		358	Yes	Yes
3	150	0.44	0.28		0.1		0.05			226		1.25		580	Yes	Yes
4	138	9.7	9.0		0.4		<0.01			337		1.52		194	Yes	Yes
5	78	16.34	15.62		0.39		<0.01			347		24.37		373	Yes	Yes
6	167	18.56	17.37		0.87		0.02			55		0.14		280	Yes	Yes
7	316	5.64	4.9		0.46		<0.01			28		0.17		382	Yes	Yes
8	120	5.93	4.23		0.7		0.80			30		0.51		178	Yes	Yes
9	73	8	6.4		0.6		0.31			177		0.21		308	Yes	Yes
10	210	11	8.61		1.28		<0.01			79		0.51		454	Yes	Yes
11	90	16.19	14.4		1.3		<0.01			20		1.12		2504	Yes	Yes
12	147	7.97	6.79		0.33		<0.01			35		1.97		238	Yes	Yes
13	248	2.9	2.2		0.4		0.01			115		2.17		412	Yes	Yes
14	208	11.9	9.7		1.6		0.09			89		0.11		192	Yes	Yes
15	350	8.47	6.91		1.11		0.06			14		0.09		336	Yes	Yes
16	90	4.21	2.82		0.74		0.27			43		<0.05		293	Yes	Yes
17	120	19.2	17.28		0.86		<0.01			118		3.12		760	Yes	Yes

WBC, white blood cell; CRP, C-reactive protein; LDH, lactate dehydrogenase.

### Microbiological findings and clinical implications

**Figure 1:**
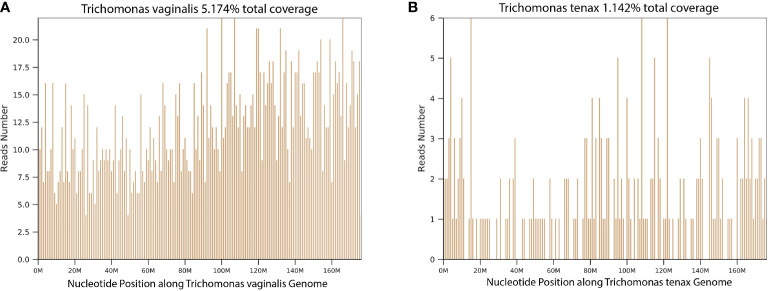
The coverage of Trichomonads vaginalis and Trichomonads tenax in the mNGS results. **(A)** Trichomonads vaginalis (Case 1); **(B)** Trichomonads tenax (Case 4).

In this cohort, *T. tenax* and *T. vaginalis* were detected in BALF samples of 15 and 2 patients, respectively. The coverage of *T. vaginalis* and *T. tenax* in the metagenomic next-generation sequencing (mNGS) results of two representative cases is illustrated in **Figure 1**, indicating the reliable detection of trichomonads. Co-pathogens identified in BALF included bacteria (10 patients), fungi (4 patients), and viruses (6 patients). Notably, two out of 17 patients (Case 4 and 17) had trichomonads as the sole microbes detected in BALF by mNGS. PCR confirmation using the same BALF samples was conducted, and all showed positive detections of *T. tenax* or *T. vaginalis*. The total number of reads, number of reads mapped on reference genomes, number of reads mapped on the human genomes and specific number of reads at the species level are all listed in **Supplementary Table 1**.

As indicated in **Table 2**, patients were categorized into two groups based on the presence of risk factors for trichomonad infection, as described in the methods. Among the 11 patients with risk factors (Case 1-11), 4 had immunocompromised conditions, 6 had uncontrolled diabetes mellitus, and 1 had aspiration due to cerebral hemorrhage. Four out of the 11 patients received nitromidazoles as either the first or second-line treatment, all experiencing relief, resulting in a 100% treatment success rate. Strikingly, among the 7 patients with risk factors who did not receive nitromidazoles, only one achieved relief despite receiving broad-spectrum and targeted antibiotic therapy based on mNGS and other microbial test results, leading to a low treatment success rate of 14.3%. Among patients without risk factors for trichomonad infection (Case 12-17), none received nitromidazoles during hospitalization, and 4 out of 6 (66.7%) eventually experienced relief.

**Table 2:** Microbiological findings and clinical outcomes of patients.

Case No.	Trichomonads species	Other microbes identified by mNGS	PCR confirmation	With risk factors	Efficacy of initial antibiotic treatment	Use of nitromidazoles	Clinical outcomes
1	*T. vaginalis*	*P. jirovecii, HSV1*	Positive	Yes	Failed	Second line	Relieved
2	*T. tenax*	*S. pneumoniae, H. influenzae, S.aureus*	Positive	Yes	Failed	Second line	Relieved
3	*T. vaginalis*	*H. parainfluenzae, C. albicans, HSV1*	Positive	Yes	Failed	Second line	Relieved
4	*T. tenax*	*None*	Positive	Yes	Succeeded	First line	Relieved
5	*T. tenax*	*L. pneumophila, Tropheryma whipplei*	Positive	Yes	Succeeded	No	Relieved
6	*T. tenax*	*A. fumigatus*	Positive	Yes	Failed	No	Death
7	*T. tenax*	*Stenotrophomonas maltophilia*	Positive	Yes	Failed	No	Death
8	*T. tenax*	*Burkholderia cepacia*	Positive	Yes	Failed	No	Death
9	*T. tenax*	*A. baumanii, Tropheryma whipplei*	Positive	Yes	Failed	No	Death
10	*T. tenax*	*A. baumanii, K.pneumoniae*	Positive	Yes	Failed	No	Death
11	*T. tenax*	*Candida albicans, HSV1*	Positive	Yes	Failed	No	Death
12	*T. tenax*	*K.pneumoniae*	Positive	No	Failed	No	Relieved
13	*T. tenax*	*K.pneumoniae, EBV*	Positive	No	Failed	No	Relieved
14	*T. tenax*	*A. baumanii, S.aureus*	Positive	No	Succeeded	No	Relieved
15	*T. tenax*	*Human herpesvirus 7*	Positive	No	Succeeded	No	Relieved
16	*T. tenax*	*P. jirovecii, HSV1*	Positive	No	Failed	No	Death
17	*T. tenax*	*None*	Positive	No	Failed	No	Death

T. tenax, Trichomonas tenax; T. vaginalis, Trichomonas vaginalis; mNGS, metagenomic next-generation sequencing; PCR, polymerase chain reaction; IMV, invasive mechanical ventilation; H. parainfluenzae, Haemophilus parainfluenzae; C. albicans, Candida albicans; HSV1, Herpes simplex virus 1; S. pneumoniae, Streptococcus pneumoniae; H. influenzae, Haemophilus influenza; S.aureus, Staphylococcus aureus; K.pneumoniae, Klebsiella pneumoniae; EBV, Epstein-Barr Virus; A. baumanii, Acinetobacter baumannii; L. pneumophila, Legionella pneumophila; A. fumigatus, Aspergillus fumigatus.

### Description of case 1

**Figure 2:**
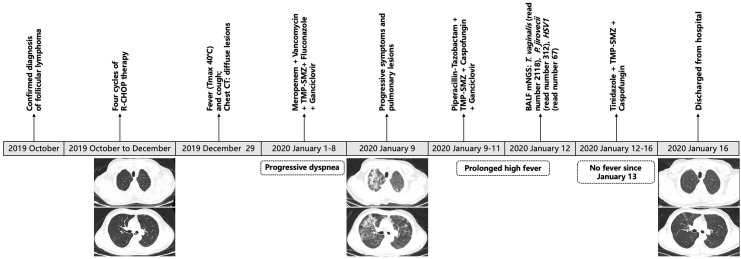
The disease course of a patient (Case 1) who was diagnosed with pulmonary trichomoniasis. R-CHOP, a combination chemotherapy including rituximab, cyclophosphamide, doxorubicin hydrochloride (hydroxydaunorubicin), vincristine sulfate (Oncovin), and prednisone; Tmax, maximum body temperature; TMP-SMZ, trimethoprim-sulfamethoxazole; mNGS, metagenomic next-generation sequencing; *HSV1*, *Herpes simplex virus 1*.

The course of illness of Case 1 was shown in **Figure 2**. This 63-year-old man was diagnosed with follicular lymphoma in October 2019 and received 4 cycles of R-CHOP therapy, a standard combination chemotherapy for non-Hodgkin lymphoma, from October to December 2019. A chest tomography on December 20, 2019, showed no apparent abnormality in the bilateral lungs. On January 1, 2020, the patient began to present with fever, cough, and dyspnea, and his chest tomography showed diffuse pulmonary lesions. After comprehensive and strong antimicrobial therapy with a combination of meropenem, vancomycin, trimethoprim-sulfamethoxazole, fluconazole, and ganciclovir, his symptoms still deteriorated. Therefore, the patient was admitted to our hospital on January 9, 2020. He had a periodontal disease but did not report any history of oral sex. Piperacillin-tazobactam, trimethoprim-sulfamethoxazole, caspofungin, and ganciclovir were empirically used for 72 hours. However, his symptoms of high fever and dyspnea did not relieve. A bronchoscopic examination was performed, and the mNGS result revealed *T. vaginalis* (specific read number 2118), *Pneumocystis jirovecii* (specific read number 312), *Herpes simplex virus 1* (specific read number 67) in the BALF sample on January 12. Subsequently, tinidazole was given against the infection of *T. vaginalis*, and trimethoprim-sulfamethoxazole combined with caspofungin were continued against *Pneumocystis jirovecii*. His body temperature fell to the normal range within 48 hours. On January 16, chest tomography showed that pulmonary infiltrates were completely absorbed, and then the patient was discharged.

## Discussion

The prevalence of pulmonary trichomoniasis remains uncertain, primarily attributed to the challenge of recognizing this disease. This difficulty arises from insensitive microbial methods and the absence of specific clinical features. Recently, the identification of *T. tenax* and *T. vaginalis* in BALF by mNGS has garnered attention, yet its clinical implications remain unclear. In this retrospective multicenter study, our data suggests that trichomonad infection should be considered in patients with identified risk factors. Consequently, we provide practical suggestions for cases where trichomonads are detected in BALF by mNGS. These findings contribute to enhancing awareness and understanding of trichomoniasis in the context of pulmonary diseases.

Over the last two decades, various trichomonad species have been identified in human lungs through immunological and molecular methods, including *T. tenax* (Duboucher et al., 2003), *T. vaginalis* (Duboucher et al., 2003; Mallat et al., 2004), and *Pentatrichomonas hominis* (Jongwutiwes et al., 2000), which suggests that human trichomonad infections may not be restricted to a specific anatomic site (Maritz et al., 2014). Trichomonads can also invade the pleura and cause empyema (Lewis et al., 2003; Mantini et al., 2009). However, the epidemiology, pathophysiology, clinical features, and management options of pulmonary trichomoniasis remain largely unclear (Duboucher et al., 2008; Martínez-Girón et al., 2008). Due to a lack of recognition of this disease, the possibility of trichomonad infection in human lungs has often been ignored among patients with suspected pneumonia. Exclusive reliance on empirical antibiotic treatment does not cover trichomonads, leading to a worse prognosis in patients with pulmonary trichomoniasis. Therefore, an efficient microbial method for detecting trichomonads is urgently needed in clinical practice.

Currently, the most common approach for detecting trichomonads in hospitals is through microscopic examination of staining smears. However, its low sensitivity is attributed to numerous factors (Dos Santos et al., 2017). The immobility of trichomonads due to low temperature or extended periods *in vitro*, the similarity in movement between many epithelial cells of lung alveoli and trichomonads, and the ability of trichomonads to develop into an unrecognizable amoeboid form contribute to this low sensitivity (Duboucher et al., 2007a; Li and Gao, 2016). BALF samples are considered diluted, further reducing the sensitivity of trichomonad detection. PCR emerges as an alternative effective tool due to its high sensitivity and specificity (Lin et al., 2019). Although the advantages of PCR have been confirmed through direct comparisons in both vaginal and BALF samples (Heine et al., 1997; Lawing et al., 2000; Lin et al., 2019), routine use of PCR assays targeting trichomonads in pneumonia patients is impractical for physicians due to a lack of awareness and limited knowledge related to pulmonary trichomoniasis. Moreover, PCR assays for trichomonads are unavailable at most hospitals. In our present study, the data indicate that mNGS enables efficient detection of trichomonads in BALF samples. Its advantages, including being untargeted and high-throughput, make it a valuable tool, and the results can be validated by PCR assays. The application of mNGS provides crucial guidance for antimicrobial treatment. Recently, metatranscriptomics emerges as a valuable and complementary approach to metagenomics in studying infectious diseases. It analyzes the total RNA content of a sample, revealing which genes are actively being transcribed by the microbial community. Therefore, metatranscriptomics offers insights into the gene expression profiles of microorganisms within a sample, providing information about actively transcribing genes. This can be particularly relevant in understanding the dynamics of microbial activity, such as during active infections (Adekoya et al., 2023; Wang et al., 2023).

**Figure 3:**
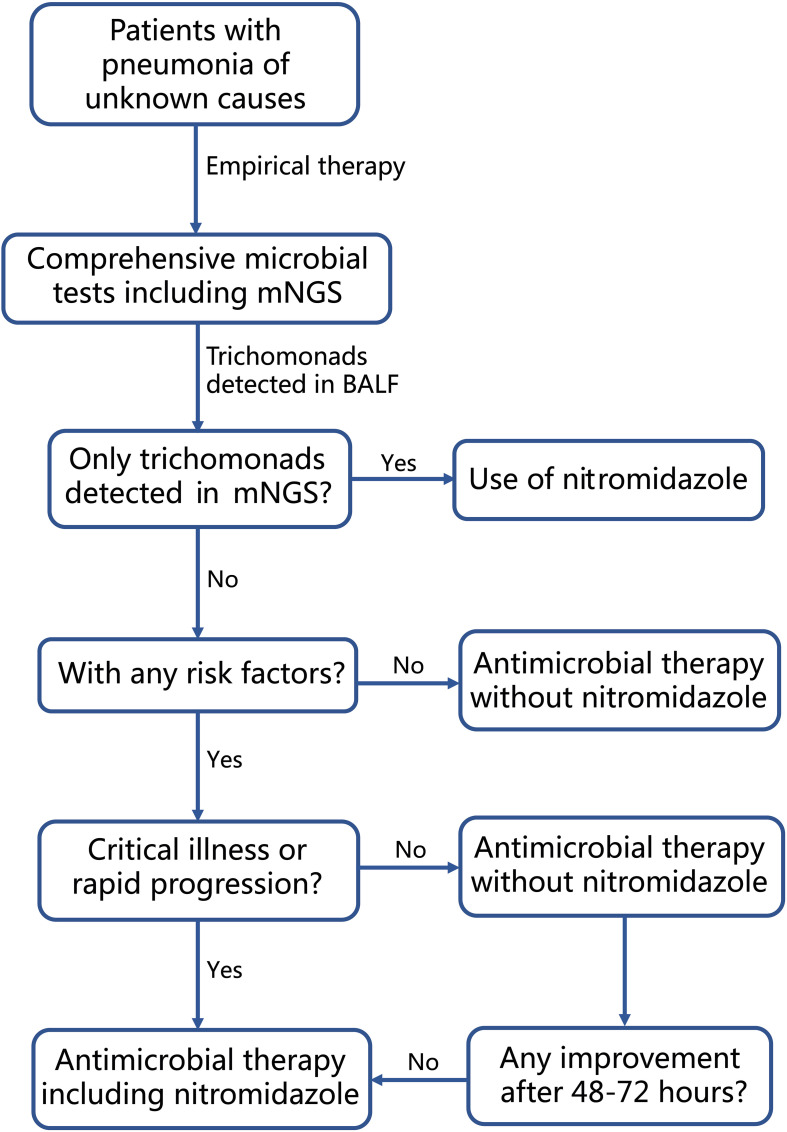
Flow-chart for clinical management of patients diagnosed with pneumonia and having trichomonads detected in BALF by mNGS.

When advanced molecular methods, such as mNGS and PCR, are employed to detect trichomonads in respiratory tract specimens, a crucial question arises: how to distinguish infection from contamination and colonization? In this study, *T. tenax* and *T. vaginalis* were detected in BALF samples of 15 and 2 patients, respectively. As a common obligate symbiont in the human mouth, especially in patients with poor oral hygiene, the presence of *T. tenax* in the human respiratory tract may be attributed to the aspiration of the parasite from the oral cavity, potentially not leading to pulmonary infections. Hence, oral or periodontal diseases and aspiration could be considered risk factors for *T. tenax* infection. Previous literature has indicated that various degrees of immunosuppression, uncontrolled diabetes mellitus, dysphagia and alcohol abuse are common comorbidities in patients with trichomonad infections, including *T. tenax* and *T. vaginalis* (Duboucher et al., 2007b; Leterrier et al., 2012; Lopez-Escamilla et al., 2013; Dybicz et al., 2018). Consequently, we categorized the 17 patients into two groups based on the presence or absence of these risk factors. Among the 11 patients with risk factors (4 with immunocompromised conditions, 6 with uncontrolled diabetes mellitus, 5 with oral or periodontal diseases, and 1 with aspiration due to cerebral hemorrhage), the administration of nitromidazoles correlated with better outcomes, resulting in a higher treatment success rate (4/4 vs. 1/7). In contrast, among the 6 patients without risk factors, none received nitromidazoles, yet 4 showed relief. This suggests that *T. tenax* detected in these patients could be either colonization or contamination during bronchoscopy. This observation reinforces the identification of risk factors for trichomonad infection and supports the use of nitromidazoles as salvage therapy when the first-line treatment fails in patients with these risk factors and trichomonads detected in BALF. For critically ill patients with rapidly progressive pneumonia, we propose the inclusion of nitromidazoles as a component of the first-line therapy to prevent delayed treatment against trichomonads. In cases where trichomonads are the sole microbes detected in BALF by mNGS, as seen in Case 4 and Case 17 in our cohort, the use of nitromidazoles is recommended. Based on our data and clinical experience, we developed a clear guiding strategy flowchart for the clinical management of patients with trichomonads detected in BALF, as shown in **Figure 3**.

It is noteworthy that two patients (Case 1 and 3) had *T. vaginalis* detected in BALF samples in this study. According to existing knowledge, *T. vaginalis* should not exist in healthy human lungs, except for its identification in the pharynx and lower respiratory tract of neonates born to mothers with *T. vaginalis* infection and adults with a history of urogenital contact (Salvador-Membreve et al., 2014). Both Case 1 and 3 were immunocompromised patients with hematological malignancies, received nitromidazoles, and eventually experienced relief. Although the invasion pathway and pathogenicity of *T. vaginalis* remain unclear, our data suggest that close attention should be paid when *T. vaginalis* is detected in BALF samples.

The co-occurrence of trichomonads and *Pneumocystis jirovecii* in the lungs is a compelling area of research, especially in immunocompromised individuals. While both pathogens can independently affect the respiratory system, their potential interaction within the lung environment has attracted attention in clinical settings. Research has indicated that trichomonads are frequently found in the course of human *Pneumocystis jiroveci* pneumonia (Duboucher et al., 2007b). The hypothesis is that local alveolar conditions, rather than immunodepression, are the main factors favoring the development of trichomonads (Duboucher et al., 2008). However, in our present study, only two cases (Case 1 and 16) showed trichomonads and *Pneumocystis jirovecii* simultaneously detected in BALF samples. Furthermore, a previous study conducted by our team showed that trichomonads were barely detected in BALF from 60 patients with *Pneumocystis jirovecii* pneumonia (Jiang et al., 2021). Therefore, the precise relationship between trichomonads infections in the lungs and co-infection with *Pneumocystis jirovecii* remains relatively understudied and not well understood. Further epidemiological studies are needed to understand the prevalence, risk factors, and clinical outcomes associated with the co-infection of trichomonads and *Pneumocystis jirovecii* in immunocompromised individuals.

The present study has limitations. Firstly, it is a retrospective study, and only a small number of patients are included due to the low detection rate of trichomonads in BALF. Because of its retrospective nature, microscopic examination of staining smears was not performed to further confirm the presence of trichomonads. Multicenter studies with a larger sample size are required to confirm our findings and provide physicians with more comprehensive guidance when dealing with trichomonads in BALF. Secondly, there are no widely accepted diagnostic criteria for pulmonary trichomoniasis so far, and physicians must recognize that trichomonads detected in BALF by mNGS cannot definitively indicate infection. A comprehensive analysis of clinical, laboratory, and radiological characteristics, as well as the response to empirical antimicrobial treatment, is necessary for the diagnosis of pulmonary trichomoniasis. In this study, several samples have very low number of reads mapping onto Trichomonas genomes (such as Case 12 and 14), which suggests the possibility of contamination either in the sample or on the sequencing platform. Therefore, accurate interpretation of mNGS results is crucial when clinical physicians use this technology.

## Conclusion

The prevalence, characteristics, and impact of trichomonads in the lungs deserve much more attention. While mNGS is an effective tool for detecting trichomonads in BALF of patients with pneumonia, it cannot distinguish between infection, colonization, and contamination. Trichomonad infection should be considered if detected in BALF samples of patients with risk factors. When broad-spectrum antibiotics targeting other coexisting pathogens fail, nitroimidazoles are recommended against trichomonads, which could potentially improve prognosis. Further investigations are required to understand the significance of the pulmonary settlement of trichomonads, their real incidence, invasion pathway, and pathogenicity.

## Data availability statement

The datasets presented in this study can be found in online repositories. The names of the repository/repositories and accession number(s) can be found below: https://db.cngb.org/cnsa/, CNP0005055, CNR1065172, CNR1065173, CNR1065174, CNR1065187, CNR1065188, CNR1065189, CNR1065190, CNR1065191, CNR1065192, CNR1065193, CNR1065194, CNR1065195, CNR1065196, CNR1065197, CNR1065198, CNR1065199, CNR1065200.

## Ethics statement

The studies involving humans were approved by the Institutional Review Board and Ethics Committee of Central South University. The studies were conducted in accordance with the local legislation and institutional requirements. The ethics committee/institutional review board waived the requirement of written informed consent for participation from the participants or the participants’ legal guardians/next of kin because this is a retrospective study and all research data were de-identified and anonymously analyzed.

## Author contributions

JJ: Data curation, Formal Analysis, Funding acquisition, Investigation, Methodology, Writing – original draft. YL: Conceptualization, Data curation, Investigation, Validation, Writing – review & editing. QW: Investigation, Resources, Writing – review & editing. HZ: Investigation, Resources, Writing – review & editing. WY: Investigation, Writing – review & editing. YW: Investigation, Writing – review & editing. WP: Investigation, Writing – review & editing. PP: Funding acquisition, Supervision, Writing – review & editing. CH: Funding acquisition, Supervision, Writing – review & editing. PD: Conceptualization, Data curation, Formal Analysis, Methodology, Project administration, Resources, Supervision, Validation, Visualization, Writing – original draft.
