# Supplementary material for: Clinical implications of trichomonads detected in bronchoalveolar fluid by metagenomic next-generation sequencing: a multicenter retrospective study
Source: Front Cell Infect Microbiol. 2024 Jan 22;14:1289231. doi: 10.3389/fcimb.2024.1289231 (PMC10839053; doi:10.3389/fcimb.2024.1289231)
Supplement: Supplementary file 1 [file Table_1.docx]

**Supplementary Table 1. Read numbers of each bronchoalveolar fluid sample detected by mNGS**

| Case No. | Total number of reads | Number of reads mapped on reference genomes | Number of reads mapped on the human genomes | Specific read number at the species level |  |
| --- | --- | --- | --- | --- | --- |
| 1 | 48,716,667 | 748,462 | 47,810,537 | *T. vaginalis* (2,118), *P. jirovecii* (312)*, HSV1* (67) |  |
| 2 | 38,244,944 | 12,948,227 | 8,720,559 | *T. tenax* (55), *S. pneumoniae* (15,8552)*, H. influenzae* (456)*, S.aureus* (374) |  |
| 3 | 33,160,716 | 7,054,030 | 25,205,460 | *T. vaginalis* (110), *H. parainfluenzae* (336,898)*, C. albicans* (195)*, HSV1* (3,397) |  |
| 4 | 44,082,052 | 458,479 | 2,995,761 | *T. tenax* (10,788) |  |
| 5 | 101,242,204 | 145,595 | 92,484,549 | *T. tenax* (110), *L. pneumophila* (2,001)*, Tropheryma whipplei* (32) |  |
| 6 | 46,893,401 | 4,282,601 | 35,798,421 | *T. tenax* (65), *A. fumigatus* (2,650,097) |  |
| 7 | 37,582,239 | 45,138 | 34,344,260 | *T. tenax* (94), *Stenotrophomonas maltophilia* (12,856) |  |
| 8 | 144,596,428 | 1,543,446 | 127,239,904 | *T. tenax* (89), *Burkholderia cepacian* (74) |  |
| 9 | 102,217,991 | 84,599 | 93,245,599 | *T. tenax* (87), *A. baumanii* (495)*, Tropheryma whipplei* (240) |  |
| 10 | 21,071,357 | 950,278 | 19,988,289 | *T. tenax* (595), *A. baumanii* (8,175)*, K.pneumoniae* (962) |  |
| 11 | 56,264,834 | 3,341 | 51,050,180 | *T. tenax* (28), *Candida albicans* (5,684)*, HSV1* (335) |  |
| 12 | 44,713,500 | 432,706 | 43,331,931 | *T. tenax* (7), *K.pneumoniae* (147,003) |  |
| 13 | 46,907,039 | 400,346 | 43,036,095 | *T. tenax (20)*, *K.pneumoniae* (1,225)*, EBV* (40) |  |
| 14 | 24,064,515 | 3,273,828 | 15,420,602 | *T. tenax* (5), *A. baumanii* (1,526,344)*, S.aureus* (129,961) |  |
| 15 | 66,258,187 | 2,600,745 | 47,473,342 | *T. tenax* (20), *Human herpesvirus 7* (34) |  |
| 16 | 126,483,281 | 31,737 | 114,472,564 | *T. tenax* (152), *P. jirovecii* (4)*, HSV1* (74) |  |
| 17 | 55,406,704 | 659,535 | 48,171,052 | *T. tenax* (614) |  |

mNGS, metagenomic next-generation sequencing; SMRN, stringently mapped read number;*T. tenax*, *Trichomonas tenax*; *T. vaginalis*, *Trichomonas vaginalis*; mNGS, metagenomic next-generation sequencing; PCR, polymerase chain reaction; IMV, invasive mechanical ventilation; *H. parainfluenzae*, *Haemophilus parainfluenzae*; *C. albicans*, *Candida albicans*; *HSV1*, *Herpes simplex virus 1*; *S. pneumoniae*, *Streptococcus pneumoniae*; *H. influenzae*, *Haemophilus influenza*; *S.aureus*, *Staphylococcus aureus*; *K.pneumoniae*, *Klebsiella pneumoniae*; *EBV*, *Epstein-Barr Virus*; *A. baumanii*, *Acinetobacter baumannii*; *L. pneumophila*, *Legionella pneumophila*; *A. fumigatus*, *Aspergillus fumigatus*.
